# Sentiment classification for employees reviews using regression vector- stochastic gradient descent classifier (RV-SGDC)

**DOI:** 10.7717/peerj-cs.712

**Published:** 2021-09-23

**Authors:** Babacar Gaye, Dezheng Zhang, Aziguli Wulamu

**Affiliations:** School of Computer and Communication Engineering, University of Science and Technology, Beijing, China

**Keywords:** Employees classification, Machine learning, Sentiment analysis, Hybrid model, Text classification

## Abstract

The satisfaction of employees is very important for any organization to make sufficient progress in production and to achieve its goals. Organizations try to keep their employees satisfied by making their policies according to employees’ demands which help to create a good environment for the collective. For this reason, it is beneficial for organizations to perform staff satisfaction surveys to be analyzed, allowing them to gauge the levels of satisfaction among employees. Sentiment analysis is an approach that can assist in this regard as it categorizes sentiments of reviews into positive and negative results. In this study, we perform experiments for the world’s big six companies and classify their employees’ reviews based on their sentiments. For this, we proposed an approach using lexicon-based and machine learning based techniques. Firstly, we extracted the sentiments of employees from text reviews and labeled the dataset as positive and negative using TextBlob. Then we proposed a hybrid/voting model named Regression Vector-Stochastic Gradient Descent Classifier (RV-SGDC) for sentiment classification. RV-SGDC is a combination of logistic regression, support vector machines, and stochastic gradient descent. We combined these models under a majority voting criteria. We also used other machine learning models in the performance comparison of RV-SGDC. Further, three feature extraction techniques: term frequency-inverse document frequency (TF-IDF), bag of words, and global vectors are used to train learning models. We evaluated the performance of all models in terms of accuracy, precision, recall, and F1 score. The results revealed that RV-SGDC outperforms with a 0.97 accuracy score using the TF-IDF feature due to its hybrid architecture.

## Introduction

In recent years, people have adopted new ways of communication and interaction with each other due to the development of information and communication technologies and the exponential growth of Word Wide Web applications. People communicate their experiences, opinions, interests, etc. on online platforms such as Facebook, Twitter, Reddit, and other web forums provided by organizations for employees. Employee online reviews are marked as the most prevalent form of user-generated content which has the potential to convey critical and useful meanings to their readers along with the satisfaction of employees of the company’s services. Other than social media platforms, many companies such as Samsung, Microsoft, Netflix, etc. provide a platform for their employees to express their personal opinions regarding the company’s policies, working environment, and products. Considering the reviews and comment assists in decision-making processes for business owners to redesign policies or make changes to products, it is important to have the means to collect data effectively. Similarly, employee online reviews regarding their job experience are a major contribution in defining a company’s reputation for its employee-centeredness positivity, and supportiveness. Being the voice of employees, mining the sentiment of these reviews offers new opportunities for company owners and managers to acquire valuable insights by the satisfaction and dissatisfaction of employees with their job (Feng, 2020). The main aim of this research is to help organizations to make good progress in production. Organizations try to keep their employees happy and make their policies according to employees’ demands which help to create a good environment between the organization and employees. The proposed research is based on Sentiment Classification for Employees Reviews and categorized into positive or negative reviews so the organization can easily find the satisfaction or dissatisfaction of employees about their products.

Sentiment analysis (SAs) is a natural language processing (NLP) task which deals with the computation of people’s emotions, opinions, and sentiments directed towards a specific entity (Basiri et al., 2021). Different studies (Medhat, Hassan & Korashy, 2014) used sentiments analysis to solve different problems. SAs aims to reveal the positive or negative sentiment in data examined and, in some cases, neutral sentiment can also be considered which was not the case in this study as we consider this study as a binary classification problem. There are several relevant methods in SAs that can be integrated in analyzing sentiments of employees’ reviews, lexicon-based method, and supervised machine learning method (Özçelik et al., 2021). The machine learning method requires annotated data for training which is often difficult to acquire (Huang et al., 2020). Comparatively, the lexicon-based method utilizes a pre-defined dictionary, also known as, sentiment lexicon for exploring sentiments of data under analysis (Khan et al., 2021b). This study utilizes a dataset in which employees’ reviews are unannotated, making the lexicon-based method more appropriate for the current analysis.

A sentiment lexicon is a database of sentiment bearing words with their corresponding polarity scores (Mohamed, Moussa & Haggag, 2020). A sentiment lexicon works by assigning respective polarity score (*P*_*s*_*core*) to the sentiment bearing words (*W*_*n*_) in a whole text, which is further aggregated into an overall polarity score (Overall ${P}_{s}core={\mathop{\sum }\nolimits }_{i=1}^{n}{P}_{(scor{e}_{n})}$). Each sentiment lexicon has a relative overall polarity score range(*R*_(*P*_*s*_*core*)_ = [*R*_+_, *R*_−_]) in which *R*_+_ corresponds to the positive sentiment and *R*_−_ corresponds to negative sentiment value. The resulting overall polarity score is then compared with a threshold value (th), explicitly set by the programmer, which discloses the nature of the text under analysis.

 
_______________________ 
Algorithm 1 Algorithm to find sentiments______________________________________________ 
Input: Text under analysis (W _n) 
Output: Sentiment 
Assigning Polarity Score (P _score) 
W _1 ⇐= P _score_1 
W _2 ⇐= P _score_2 
W _n ⇐= P _score_n 
Aggregating Polarity Score 
Overall P _score = ∑ 
  W _1 + W _2 + +W _n 
Comparing with Threshold Value 
if 
 Overall P _score > 0 
return 
  Sentiment (Positive) 
else-if 
 Overall P _score < 0 
return 
  Sentiment (Negative)__________________________________________________________________________    

The foundation of this study is that utilizing polarity-based sentiments of employees’ online reviews as the ground truth for prediction of job satisfaction or dissatisfaction, produces more accurate results as compared to previous studies (Rustam et al., 2021a; Rehan et al., 2021). For this purpose, the current study incorporates preexisting SLs for exploiting the sentiment score of employees’ reviews which are further classified by machine learning (ML) classifiers. In addition to this, a voting classifier is proposed and evaluated on employees’ reviews dataset containing employee reviews from Facebook, Google, Microsoft, Apple, and Amazon (Rehan, 2020).

The main contributions for this study are listed below:

• TextBlob is utilized for extracting the sentiment score of the reviews which are further categorized as positive and negative sentiments based on the threshold value. These sentiments are incorporated as the ground truth for further analysis in this study.
• Seven supervised machine learning algorithms including logistic regression (LR), random forest (RF), AdaBoost Classifier (AC), Multi-layer Perceptron (MLP), Stochastic Gradient Descent Classifier (SGDC), Support Vector Classifier (SVC), and Extra Tree Classifier (ETC) are examined to evaluate their performance on a dataset containing employee reviews.
• A voting classifier referred to as Regression Vector Stochastic Gradient Descent Classifier (RV-SGDC) is devised to classify employees’ reviews as positive and negative. RV-SGDC is based on LR, SVC, and SGDC under hard voting. The performance of the proposed voting classifier is compared with selected state-of-the-art ML models.
• The effficacy of three feature extraction techniques including term frequency-inverse document frequency (TF-IDF), bag of words (BoW), and global vectors for word representation (GloVe) is compared.
• Selected classification models along with a proposed voting classifier are evaluated and compared in terms of accuracy, precision, recall, and f1-score.
• Performance of results is analyzed on another data set Twitter US Airline Sentiment.
• Performance of proposed framework is analyzed against the previous study conducted concerning job satisfaction prediction which considered users’ rating as the ground truth for carrying out a sentiment analysis of employees’ reviews (Rustam et al., 2021a; Rehan et al., 2021).

The rest of the paper is structured as ‘Related work’ provides a deep insight into the previous researches carried out related to the current study. Additionally, this section describes the contribution of the present study to the literature. ‘Materials and Methods’ discusses the dataset in detail, data preparation techniques, feature extraction techniques, machine learning models, and evaluation metrics used in the proposed framework. ‘Proposed Approach’ describes the proposed architecture in detail. ‘Experimental Results’ explores the results of the proposed approach along with its comparison with state-of-the-art methods. This section also compares the current study with the previous study. ‘Conclusion’ concludes the study along with limitations and future work.

## Related Work

The performance of an employee is believed to be directly correlated with job satisfaction as it plays a significant role in the success and development of an organization (Kuzey, 2012). Job satisfaction is associated with a variety of variables such as higher productivity, lower turnover, and customer satisfaction. Regarding the platforms provided by organizations, employees communicate their personal opinion concerning the organization which depicts the satisfaction of employees with the organization. In recent years, researchers have more focused on exploiting employees’ reviews using machine learning models to deduce useful insights for organizations. Previous researches concerning the exploitation of job satisfaction and dissatisfaction from employees’ reviews are briefly described in Table 1.

**Table 1:** Brief description of previous work related to present study.

**Ref.**	**Summary**
Moniz & de Jong (2014)	Forecasting of organizations’ earnings is correlated with the satisfaction level of employees of an organization. As stated by the authors of this study as they identified one significant aspect of employees’ reviews using Latent Dirichlet Allocation (LDA) which was associated with the organizations’ outlook. Authors integrated the General Inquirer dictionary to explore sentiment of the reviews which was further utilized for exploring job satisfaction of employees when combined with the outlook aspect of the organization.
Costa & Veloso (2015)	Contributed to employee analytics by focusing on improving text representations by computing vector representations of fixed lengths. The authors implemented word2vec for extracting features of employees’ reviews and used SVR and SVM as the machine learning classifiers for sentiment-based classification of reviews.
Jung & Suh (2019)	Derived 30 factors and their corresponding keywords, impacting the degree of job satisfaction, using LDA. Afterward, the authors conducted importance and sentiment analysis of these factors at various levels. They calculated the sentiment based on the frequency of nouns correlated with the description of job satisfaction factors. They also analyzed the most dominant factors impacting job satisfaction.
Bajpai et al. (2019)	Proposed ELM (extreme learning machine); an ensemble model which integrated SentiWordNet and SenticNet for exploiting sentiment of the reviews and machine learning models for classifying the satisfaction and dissatisfaction of employees. In addition to this, the authors also evaluated the proposed approach using SVM. Results showed that ELM obtained a 74.09% accuracy score whereas, SVM yielded an accuracy score of 74.85%.
Stamo lampros et al. (2019)	Explored the employee turnover and determinants of job satisfaction by incorporating. They exploited factors impacting job satisfaction from employees’ reviews obtained from Glassdoor.com using an unsupervised approach of structural topic modeling such as LDA. In addition to this, the authors also explored the impact of the overall rating given by employees on the satisfaction of employees with the job. The study concluded that positive reviews are more correlated to the factors such as leadership, culture, career opportunities, etc. Contrarily, negative reviews correspond to topics such as communication with management, managerial behavior, etc.
Kashive, Khanna & Bharthi (2020)	Conducted sentiment analysis of employees’ reviews along with text analysis using SAS analyzer tool. Authors carried out their research on employees’ reviews from four sectors including info-tech, manufacturing, FMCG, and Pharmaceuticals. Sentiment analysis of reviews showed that factors concerning the job satisfaction level of an employee are social value, work-life value, interest value, economical value, brand value, development value, management value, and interest value.
Dina & Juniarta (2020)	Proposed aspect-based sentiment analysis of employees’ reviews crawled from Glassdoor.com using Webharvy web-scrapper to exploit employees’ satisfaction and dissatisfaction. Crawled reviews were then preprocessed to produce keywords using RapidMiner. Afterward, authors assigned the weights to each keyword using term frequency to which Stanford POS tagger was used to tag nouns only. Nouns were then classified based on their aspect as negative and positive.
Rajendran (2020)	Proposed a four-fold framework for analysis of employees’ reviews concerning an improvement in the performance of the delivery services industry. The proposed approach integrates employee reviews related to four delivery companies which are then preprocessed to eliminate unnecessary data and separated as positive, negative, and neutral based on the overall rating. Afterward, bigrams, trigrams are extracted which are then analyzed by SWOT technique. The study concluded that most of the negative reviews by the employee were related to health issues, quality planning of routes, etc.
Rustam et al. (2021a)	Integrated machine learning and deep learning models for classifying employee reviews into satisfied and unsatisfied. Authors referred to overall ratings (1-5) as the ground truth for carrying out classification tasks. For this purpose, they assigned rating >2.5 to satisfied class and rating <2.5 to unsatisfied class. Afterward, the preprocessed the reviews and extracted features using TF-IDF which were then utilized for the training of MLP. Evaluation of the proposed model yielded an accuracy score of 83%.
Rehan et al. (2021)	Exploited the correlation between reviews written by employees and overall ratings. The authors proposed the ERCE model which combined two modules with AND gate logic. Module-1 utilized an aggregate of all numeric ratings as the ground truth, whereas, module-2 utilized overall rating as the ground truth, both modules were trained and evaluated on the employees’ review dataset. The study concluded that 76% of reviews and ratings correlate whereas, 24% of the reviews and ratings do not correlate. In addition to this, authors also classified employees’ reviews as satisfied and unsatisfied which yielded 100% accuracy by module-1 and 79% accuracy by module-2.

### Contribution to literature

Exploring the literature review in the previous section illustrates that recent approaches used for predicting job satisfaction from employees’ reviews target the rate as the ground truth, whereas Rehan et al. (2021) concluded that overall ratings are not entirely correlated with corresponding textual reviews rendering misleading information for the organizations. The present study contributes to literature proposing an efficient system for evaluation of employees’ reviews as satisfied and unsatisfied by incorporating textual features. The primary goal is to exploit the sentiment of employees’ reviews using a sentiment lexicon which is further regarded as the target class or ground truth for the machine learning models to perform classification tasks. Another limitation in employee classification is the low accuracy score in previous studies which is a motivation for other researchers to work. Concerning all the presented works, we focused on the correlated target class and the improvement in the machine learning model’s performance.

## Materials and Methods

The proposed framework is five-fold *i.e.,* pre-processing of the reviews to eliminate unnecessary data, sentiment labeling, feature extraction, training of prediction models, and evaluating the performance of trained predictors. These steps involved in carrying out job satisfaction prediction are briefly discussed in this section.

### Dataset description

Employee reviews dataset contains reviews from Facebook, Microsoft, Netflix, Apple, Amazon, and Google employees and were obtained from Kaggle (Rehan, 2020). The dataset contains a total of 67,529 records and sixteen variables among which seven are numeric variables which include numeric and star ratings given by employees from 1 to 5, and four are text variables including summary, pros, cons, and advice to management by the reviewer employee. Rest variables correspond to information regarding the reviewer employee. These variables are discussed in Table 2. Current research work concatenates four text variables for carrying out job satisfaction prediction which is illustrated in Table 3.

**Table 2:** Description of dataset variables.

Variable	Description
Index	Index of record
Company	Name of company
Location	Company location
Date	Date on which review was written
Job-Title	Job title of employee
Summary	Summary of review written by employee
Pros	Benefits regarding employees of company
Cons	Drawbacks regarding employees of company
Advice to Management	Advice given by employee to management of company
Overall Rating	Overall rating given by employee from 1–5
Work/Life Balance Rating	Rating given by employee on balance between work and life 1–5
Culture and Values Rating	Rating given by employee on culture of the company 1–5
Career Opportunities Rating	Rating given by employee showing how many career opportunities does company provide 1–5
Senior Management Rating	Rating given by employee according to management of company 1–5
Helpful Review Count	Count of how many people found this review useful
Link to Review	Link which will redirect to the particular review

**Table 3:** Sample of data utilized for research work.

Summary	Pros	Cons	Advice to management	Review
good but i rreally dont kniow about that	i think its may be even better than any other ...	hmmmm i think all the downsides working at exc...	there is only one advice to the management tha...	good but i rreally dont kniow about that i think its may…
social media marketing	creating engaging social media strategies and...	monitoring the success of social media campaig...	assisting the company’s business development t...	social media marketing creating engaging social media …

To make a comparison another dataset is used for the experimental process. Another dataset is based on sentiments of US airlines names ”Twitter US Airline Sentiment” (Figure Eight, 2019). Twitter US Airline Sentiment dataset is based on 8473 records with 18 features.

### Data preprocessing

Preprocessing techniques are used to clean the data to increase the ML model’s efficiency. Preprocessing of the data involves eradicating unreliable, noisy, and irrelevant data which affect the performance of machine learning models (Kotsiantis, Kanellopoulos & Pintelas, 2006). The current study incorporates preprocessing steps such as:

• Tokenization: Reviews are split into tokens of single words on the criteria of white-space.
• Lowercase Conversion: Each token was converted into lowercase as ML models are case sensitive.
• Spelling Correction: Misspelled words are corrected. The spell checker is used to check the misspelled words and convert them into correct words.
• Remove Numeric: Numeric values are removed such as contact number, value, or date which is not included in another review
• Removing unnecessary data: Username and stop-words do not add meaning to current analysis thus are removed.
• Remove Punctuation: Punctuations marks are removed from data, to increase the computation time. The punctuation signs like , #, $, %, &, etc., have been removed. Removal of these signs is done using regular expressions.
• Stemming: Tokens are converted into their root forms for reducing the complexity.

Preprocessing minimizes computation overhead and significantly influences predictive accuracy (Crone, Lessmann & Stahlbock, 2006). Data preprocessing is carried out using Python NLTK libraries.

### Sentiment labeling

Sentiment refers to an individual’s feelings, point of view, or opinion towards an entity. These opinions can be expressed by writing reviews or giving numeric or star ratings. Sentiments are broadly categorized as positive or negative based on the written review or rating (Khan et al., 2021b). The current study utilizes sentiment orientation of reviews written by employees’ which is explored by sentiment lexicon namely TextBlob (Rustam et al., 2021b).

#### TextBlob

TextBlob is a well-known python library that provides sentiment orientation of the text under analysis (Chaudhri, Saranya & Dubey, 2021a). It assigns a *P*_*s*_*core* which is a float value within the range, *R*_(*P*_*s*_*core*)_ = [ + 1.0,  − 1.0], in which *P*_*s*_*core* =  + 1.0 refers to positive polarity and *P*_*s*_*core* =  − 1.0 refers to negative polarity. In addition to this, TextBlob also provides a subjectivity score which quantifies the text under analysis as factual information or a personal opinion (Chaudhri, Saranya & Dubey, 2021b). This analysis is only concerned with the polarity score thus we did not integrate the subjectivity score. The threshold for TextBlob was set to 0, thus categorizing reviews with Overall *P*_*s*_*core* > 0 as positive reviews (satisfied) and Overall *P*_*s*_*core* < 0 as negative reviews (unsatisfied). Sample of polarity score assigned by TextBlob to employees’ reviews is shown in Table 4.

### Feature extraction

ML models require pre-defined features for training to carry out predictive tasks (Peng, 2021). In order to do so, features are extracted from data by using several feature extraction techniques. Feature extraction is a method that represents the text into a set of features relative to the current analysis. In this study, three feature extraction techniques such as TF-IDF, BoW, and GloVe are used to extract features from preprocessed employees’ reviews. We select these feature extraction methods because of their importance in the literature review. These methods are mostly used by the research for text classification and have good results (Rupapara et al., 2021; Khan et al., 2021b).

**Table 4:** TextBlob score assigned to review text and their corresponding sentiments class.

Review text	Polarity score	Sentiment
pockets incompetence overall google…	−0.05	Negative
bad free food bad management…	−0.291	Negative

#### Term Frequency-Inverse Document Frequency (TF-IDF)

TF-IDF is an information retrieval algorithm that statistically measures the relevance of a term in a document (Jiang et al., 2021). It works by weighing a term t’s frequency (tf) and its inverse document frequency (idf). Term frequency refers to the number of occurrences of a term in a document and inverse document frequency refers to the significance of that term in the whole dataset (D). In this study, we used the scikit-learn library, TfidfVectorizer, in which TF-IDF assigns weight to each term t in correspondence to its significance in the review R under analysis in the following manner:

(1) \begin{eqnarray*}t\,fid\,f(t,R,D)=t\,f(t,R)\times id\,f(t,D)\end{eqnarray*}

where,

(2) \begin{eqnarray*}t\,f(t,R)=log(1+freq(t,R))\end{eqnarray*}

(3) \begin{eqnarray*}id\,f(t,D)= \frac{logN}{(d\,f+1)} .\end{eqnarray*}

#### Bag of Words (BoW)

The BoW is a well-known feature extraction technique that represents text data disregarding word order and grammatical details (Sahin, 2021). It is widely used for NLP tasks, text classification, topic modeling, and information retrieval. This approach is easy to implement. Each instance in this feature extraction technique is tokenized to construct a vocabulary then, frequency of each token in the vocabulary is calculated (Boag et al., 2021). In simple words, BoW converts text of variable lengths into a vector of fixed length thus making it easy for ML models to work with data. For the implementation of BoW in our experiments, we integrated CountVectorizer which is a sci-kit-learn library in python.

#### Global Vector Representation of Words (GloVe)

GloVe was released by a group of NLP researchers from Stanford for vector representation of words in continuous space (Li, He & Chen, 2021). It maps words into relevant space by the distance between words and their semantic similarity. The GloVe is widely used for entity name recognition, machine translation, and many other NLP tasks. It mainly works by constructing a co-occurrence matrix M, in which *M*_*i*_*j* represents the frequency of word i appearing in some context of word j, in which co-occurrence is calculated by moving a context size window over each sentence in the text. The study import GloVe embedding from ‘zeugma’ library to employ in our experiments.

### Supervised machine learning models

We have incorporated eight machine learning models including LR, RF, AC, MLP, ETC, SVC, SGDC, and RV-SGDC in our experiments. These ML models are briefly described in this section. Best hyper-parameters were used for the optimization of ML models by the hit and trial method. During tuning each time we split the dataset and change the model’s hyper-parameters values. These hyper-parameters settings are shown in Table 5.

**Table 5:** Hyper-parameter settings for supervised machine learning models.

Model	Hyper-parameter Settings
LR	random_state=100, multi_class=‘ovr’, *C* = 3
RF	n_estimators=100, random_state=50, max_depth=250
AC	n_estimators=300, random_state=50
MLP	random_state=20, max_iter=300
ETC	n_estimators=200, random_state=50, max_depth=150
SGDC	max_iter=1000, tol=1e−3
SVC	kernel=‘linear’, *C* = 1.0, random_state=500
RV-SGDC	LR, SVC, and SGDC, Voting=Hard

#### Logistic regression

LR is a statistical ML model which models a set of input features (X: input) into target variables (Y: output) by means of a sigmoid function which is an ‘S’ shaped curve and restricts Y in the range of 0 and 1 (De Cock et al., 2021). Sigmoid function *σ*:*R*⟶(0, 1) is defined as:

(4) \begin{eqnarray*}\sigma (X)= \frac{1}{1+{e}^{(-X)}} \end{eqnarray*}

where e is the base of the natural log. LR with its easy implementation produces efficient results in the case of binary classification (Rupapara et al., 2021). In our experiments, we have integrated various parameters in LR such as multi_class, random_state, and C. Multi_class is set to ovr which is the best choice for binary classification, random_state is set to 100, and C is set to 3 for strong regularization.

#### Random forest

RF is a meta estimator which utilizes the bagging method for learning patterns from data. It is an ensemble of decision trees that are built on numerous sub-samples of the dataset. It produces optimized prediction results by pooling outputs obtained from each decision tree in the ensemble (Chen et al., 2021). It controls over-fitting by adding additional randomness to the model, it does so by considering the most appropriate features from a random subset of features while splitting a node instead of utilizing the most important feature. One of the most significant tasks in the construction of a decision tree in RF is the selection of a significant attribute as a root node. For this purpose, two techniques including Gini impurity and Information Gain are used Baranauskas, Oshiro & Perez (2012). The current study involves Gini impurity as the criterion to quantify the split of a node. Gini impurity for a node can be calculated as:

(5) \begin{eqnarray*}\sum _{x=}^{N}{f}_{x}(1-{f}_{x})\end{eqnarray*}

where, *f*_*x*_ is the number of occurrences of target attribute x at a node and N is the number of unique attributes. Several hyper-parameters are utilized for tuning of RF to acquire the best results. In our experiments, we set n_estimators, which defines the number of decision trees to be built in the ensemble, to 100. The randomness of samples bootstrapped in the construction of decision trees in the forest is set to 50, and the depth of each decision tree is set to 250 as shown in Table 5.

#### AdaBoost classifier

AC, also referred to as Adaptive Boosting, is an ensemble method that reassigns weights to each classified instance of the learner and higher weights are assigned to instances that had been classified incorrectly (Wu et al., 2020). AC constructs an ensemble of learners in a sequential manner in which each successive learner is constructed from preceding learners which results in less variance and bias of the models. It employs boosting for transforming a weak learner into a strong learner by concentrating on instances that are wrongly classified by a preceding weak learner. For a given dataset {(*x*_1_, *y*_1_), …, (*x*_*n*_, *y*_*n*_)} where each instance *x*_*i*_ has a corresponding target variable *y*_*i*_*ɛ*{ − 1,  + 1}, weak learners {*k*_1_, *k*_2_, …, *k*_*m*_} are combined in the form:

(6) \begin{eqnarray*}{C}_{(t-1)}({x}_{n})={w}_{1}{k}_{1}({x}_{n})+,,,+{w}_{(t-1)}{k}_{(t-1)}({x}_{n})\end{eqnarray*}

where, *w*_1_, , , *w*_(*t*−1)_ is the weight assigned to each predicted instance and *C*_(*t*−1)_(*x*_*n*_) represents the target variable at (*t* − 1)*th* iteration which is further extended to construct a strong learner by adding another weak learner *k*_*t*_ with another weight *w*_*t*_.

(7) \begin{eqnarray*}{C}_{t}({x}_{n})={C}_{(t-1)}({x}_{n})+{w}_{t}{C}_{t}({x}_{n}).\end{eqnarray*}

The hyper-parameter setting for AC used in our experiments is shown in Table 5. We set n_estimators to 300 and random_state to 50 to acquire optimized results.

#### Multilayer perceptron

MLP classifies an input vector ′*x*′ by multiplying it with a weight ’w’ and adding bias ’b’ to it, such as, output:w ×x + b. MLP is restricted to the linear mapping of input and output variables, whereas, perceptron in MLP is capable of carrying out the classification of data which is not linearly separable (Car et al., 2020). MLP is an extended feed-forward neural network that maps a non-linear relationship (*f*:*R*^*m*^⟶*R*^*n*^) between the input layer of m dimensions and output vectors of n dimensions. Additionally, MLP consists of an arbitrary number of hidden layers in which neurons are trained by integrating the back-propagation technique. Computationally, a set of {*n*_*i*_|*n*_1_, *n*_2_, …, *n*_*m*_} input features from input layer are assigned a weight in the hidden layer such as: *w*_1_*n*_1_ + *w*_2_*n*_2_ + , , ,  + *w*_*m*_*n*_*m*_ in relation to a non-linear function (f: R → R). Current study utilizes rectified linear unit function (relu: f(x) =max(0,x)) as an activation function. The hyper-parameter setting for MLP in our experiments is shown in Table 5.

#### Extra tree classifier

ETC is an ensemble of unpruned decision trees that involves an arbitrary subset of features for splitting of nodes (Bhati & Rai, 2020). Unlike RF, it integrates whole data in the construction of a decision tree instead of bootstrapping data. It involves two main parameters such as the number of randomized input features selected at each node(K), the minimum size of sample required for splitting a node *n*_*min*_, and the number of decision trees in the ensemble (M). Decision trees in ETC are less likely to be correlated due to the randomized selection of points of the split. ETC averages predictions of decision trees in the ensemble to produce a final prediction in case of regression (Alsariera et al., 2020). This study concerns binary classification thus pertaining to majority voting of decision trees’ predictions to output a final prediction. There are several hyper-parameters involved in the tuning of ETC. In our experiments we have set n_estimators which states the number of trees in the ensemble to 200, random_state to 50, max_depth of each decision tree to 150, and Gini impurity as the criteria for node split.

#### Support vector classifier

SVC is a pattern-based classifier which utilizes linear kernel function to map input vectors *x*_*i*_ into a high dimensional vector space (Rezaeinia et al., 2019). It then creates a linear hyper-plane with the optimized margin between the target classes *y*_*i*_. The linear kernel function integrates the following mathematical computation for pattern recognition.

(8) \begin{eqnarray*}K({x}_{i},{y}_{i})={x}_{i}^{{^{\prime}}}{y}_{i}.\end{eqnarray*}

Hyper-parameters concerning SVC in this study are shown in Table 5. Regularization parameter C is set to 1.0 for strong regularization and random_state, to shuffle the data for estimating probability, is set to 500 for optimized results.

#### Stochastic gradient descent

Gradient descent (GD) is a well-known optimization technique that learns the optimized values of models’ parameters at each iteration to minimize the cost function (*c*^*f*^) (Rustam et al., 2019). SGD is a variant of GD which concerns itself with random probability (stochastic) such that, at each iteration, a single sample is selected for the training of the model (Tan et al., 2021). It requires significantly less training time for finding *c*^*f*^ of only one training sample *x*^*i*^ at each iteration to reach local minima. It does so by updating the parameters of the model for each *x*^*i*^ and corresponding target class *y*^*i*^.

(9) \begin{eqnarray*}{\theta }_{j}={\theta }_{j}-\alpha ({y}^{{i}^{{}^{{^{\prime}}}}}-{y}^{i}){x}_{j}^{i}\end{eqnarray*}

where *θ*_*j*_ is the parameter and *α* is the learning rate of the model. SGD employs several hyper-parameters which supports its performance on data under analysis. In this study, loss function hinge was selected as default with *l*2 regularization. Maximum epochs were set to 1000 and the criterion of stopping iteration was set to 1*e* − 3 as shown in Table 5.

#### Regression Vector Stochastic Gradient Descent Classifier (RV-SGDC)

This study proposed an RV-SGDC model which is a combination of three individual models LR, SVC, and SGDC. The RV-SGDC outperforms all other models because of its ensemble architecture. We combined these models using majority voting criteria which mean that individual models will give their predictions for the target class and then the most predicted class by the models will be considered as the final target class. We combined LR, SVC, and SGDC because of their better performance individually as compare to other models and these models produce a more efficient and accurate model with the hard voting combination. We can describe RV-SGDC mathematically as:

(10) \begin{eqnarray*}L{R}_{prediction}=L{R}_{trained}(Data)\end{eqnarray*}

(11) \begin{eqnarray*}SV{C}_{prediction}=SV{C}_{trained}(Data)\end{eqnarray*}

(12) \begin{eqnarray*}SGD{C}_{prediction}=SGD{C}_{trained}(Data)\end{eqnarray*}

And

(13) \begin{eqnarray*}RV-SGD{C}_{prediction}=model\{ L{R}_{prediction},SV{C}_{prediction},SGD{C}_{prediction}\} .\end{eqnarray*}

**Figure 1:**
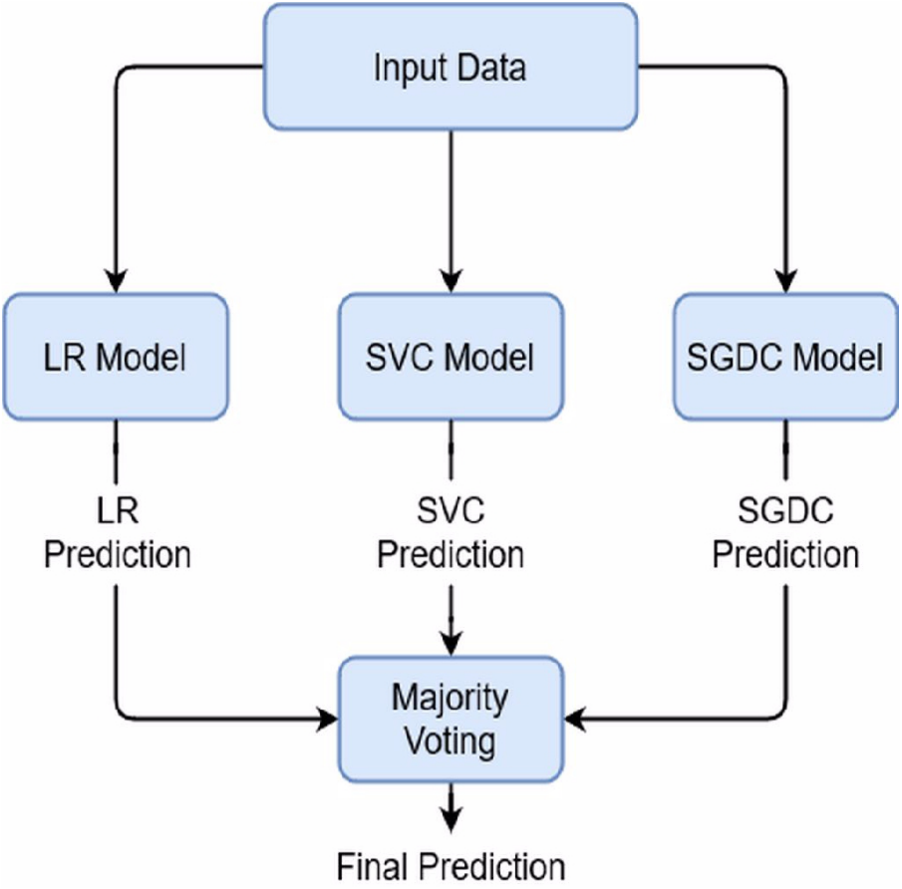
Proposed model architecture.

Here *LR*_*prediction*_, *SVC*_*prediction*_, *SGDC*_*prediction*_ are the predictions by the individual models and *RV* − *SGDC*_*prediction*_ is final prediction using majority voting between individual models’ predictions. The architecture of proposed model is shown in Fig. 1.

## Proposed Approach

The current study proposes a framework that is focused on mining sentiments of employees’ reviews. The proposed framework involves an SL for sentiment labeling and ML models for classifying employees’ reviews as positive and negative.

Employees’ reviews are first acquired by concatenating four textual features such as summary (*f*_1_), pros (*f*_2_), cons (*f*_3_), and advice to management (*f*_4_) from the dataset into a single text feature (*f*:*f*_1_ + *f*_2_ + *f*_3_ + *f*_4_). Preprocessing techniques including tokenization, lowercase conversion, numeric removal, punctuation removal, stop-word removal, and stemming were employed to remove unnecessary data for low computation. For each preprocessed f, sentiment score s is assigned using TextBlob. Then, we set threshold value to 0 which referred *s* > 0 as a positive sentiment: *f*_+_, and *s* < 0 as a negative sentiment: *f*_−_ resulting in 62,465 *f*_+_ instances and 5,064 *f*_−_ instances from a total of 67,529 records. This causes an imbalance in data which might lead to ambiguous results. Therefore, to address the problem of imbalanced data, we included 5,064 records randomly from *f*_+_ thus balancing both classes as shown in Table 6. For further process, 10128 records were used.

**Figure 2:**
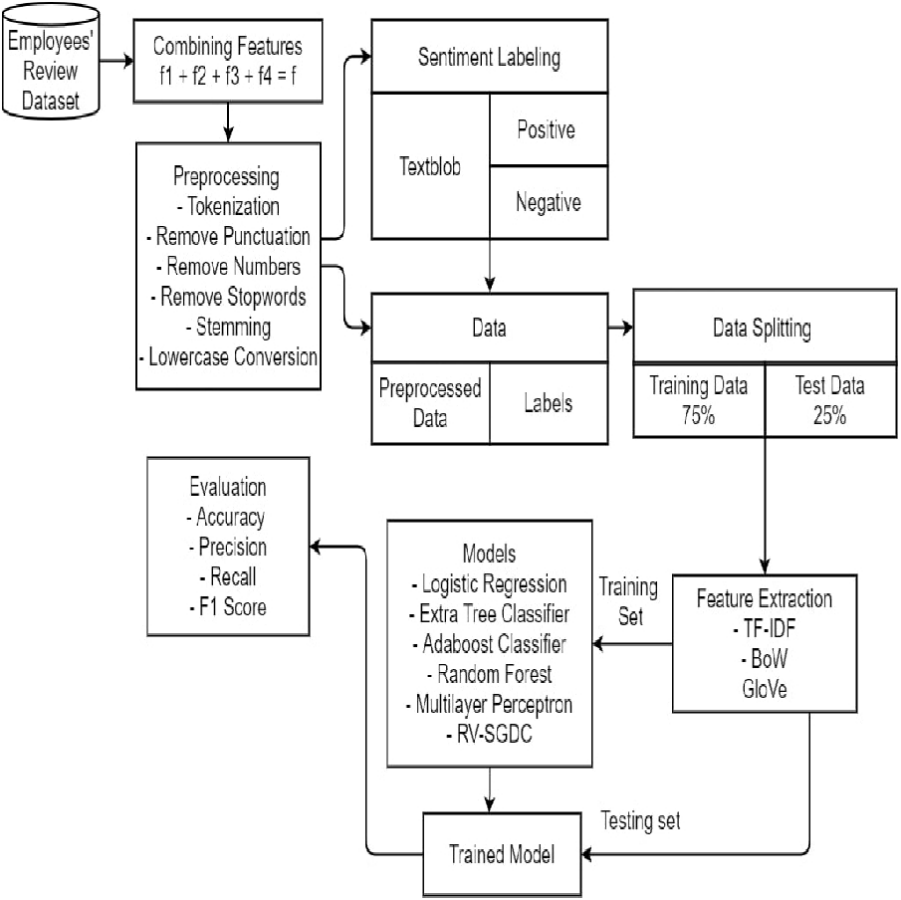
Proposed methodology.

Afterward, features were extracted from f by using TF-IDF, BoW, and GloVe. ML models and proposed RV-SGDC are then trained on feature sets extracted from feature extraction techniques and labels extracted by TextBlob. In the last, the performance of trained predictive models is evaluated in terms of accuracy, precision, recall, and f1-score on the test set. The architecture of the proposed model is illustrated in Fig. 2.

### Evaluation

Evaluation of models focuses on estimation of the performance of the model on unseen data.

Carrying out the classification of reviews into positive or negative produced four outcomes described below (Rustam et al., 2020):

**Table 6:** Dataset count for experiments.

Dataset	Positive	Negative	Total
Original dataset	62,465	5,064	67,529
Used dataset	5,064	5,064	10128

• True positive (TP): Instances that are actually positive and also predicted positive.
• True Negative (TN): Instances belonging to the negative class which are correctly predicted as negative.
• False Positive (FP): Instances that belong to negative class but are predicted positive.
• False Negative (FN): Positive instances predicted as negative instances.

• Accuracy refers to the percentage of correctly predicted instances by ML model. It can be calculated as:
  (14) \begin{eqnarray*}Accuracy= \frac{(TP+TN)}{(TP+TN+FP+FN)} .\end{eqnarray*}
• Precision refers to the percentage of predicted instances relevant to a certain class among all the instances. It can be calculated as:
  (15) \begin{eqnarray*}Precision= \frac{TP}{(TP+FP)} .\end{eqnarray*}
• Recall is the fraction of relevant instances which are successfully predicted by ML model. It can be calculated as:
  (16) \begin{eqnarray*}Recall= \frac{TP}{(TP+FN)} .\end{eqnarray*}
• F1-Score is the harmonic mean of recall and precision.
  (17) \begin{eqnarray*}F1=2\ast \frac{Precision\ast Recall}{Precision+Recall} .\end{eqnarray*}

This study evaluates the proposed approach in terms of accuracy, precision, recall, and f1-score.

## Experimental Results

This section provides details of experimental results as well as a discussion of the results. Experiments are implemented in Python language. Experiments are conducted by integrating ML models such as LR, RF, ETC, AC, SGDC, SVC, MLP, and RV-SGDC for classifying employees’ reviews into positive (satisfied) and negative (unsatisfied) classes. The efficacy of the proposed approach is tested over a 3:1 train-test set by using three different feature extraction techniques such as TF-IDF, BoW, and GloVe. The presented results of this study were achieved from sentiments, whereas the experiments were also done using the other rating attributes such as work balance, culture-values, carrier-opportunities.., as a target class but the achieved results are not as good or significant shows where the classification accuracy goes down because all these features contain the data of single departments so it is difficult to train the models whereas the review is about the whole company. Results obtained by integrating TF-IDF are shown in Table 7.

Experimental results of ML models using TF-IDF features demonstrate that LR, SVC, and SGDC perform well with a maximum accuracy score of 0.96. In terms of positive and negative classes, these three models have also yielded the highest precision, recall, and f1-score of 0.96 in comparison to other ML models. Except for SVC which yielded 0.95 precision in prediction of negative class and 0.95 recall in the prediction of the positive class. LR works best for problems on binary classifications due to its powerful working architecture involving sigmoid functions. In our experiments, LR worked well because instead of making presumptions about the categorization of positive and negative classes in vector space, it integrates maximum likelihood for the estimation of accurate results. On the other hand, SVC provides optimum generalizability to the proposed approach with minimization of structural risk along with exploiting a maximum boundary between target classes. Whereas, SGD with its capability of computing any sample at a time and frequently updating parameters assist in better learning of data under consideration and thus producing better results. Table 8 shows results of ML models obtained when trained and tested on features extracted by BoW.

Empirical results show that ML models have shown better performance with BoW features. MLP, SVC, SGDC, and AC have yielded maximum accuracy of 0.96 while ETC and RF acquired a 0.94 accuracy score on test data. Whereas, the performance of LR was not affected by changes in the feature set as can be observed by the results. TF-IDF produces weighted features which as a result reduces the feature size, on the other hand, BoW extract features regardless of their weight in the document thus creating a larger feature set. This is leveraged by MLP, since, being a neural network, it requires a larger feature set for better training. Therefore, producing better results with the BoW feature set when evaluated on the test set. Similarly, AC has shown outstanding performance as well in classifying data into positive and negative classes. Although RF and AC are both ensembles of decision trees the main difference between them is that RF assigns equal weight to decisions made by each decision tree, whereas, in AC the errors made by the preceding decision tree influence the weight-age given to the successive decision tree. This results in minimization of error and improvement in accuracy results of AC rather than RF. Consequently, ETC being a meta estimator adds randomization in choosing the split of nodes instead of selecting the optimized one. This causes low accuracy results as shown by the experiments in this study.

GloVe generates word embedding in a vector space by measuring the distance between words, but sentiment information of words is ignored by this feature extraction technique (Rezaeinia et al., 2019). For instance, it might create a co-occurrence between words good and bad which limits its performance in sentiment analysis. Results with GloVe features reveal a drop in classification accuracy of ML models in classifying employees’ reviews as shown in Table 9.

**Table 7:** Accuracy, precision, recall and F1-score results of all ML models using TF-IDF.

Model	Accuracy	Negative class			Positive class		
		Precision	Recall	F1-Score	Precision	Recall	F1-Score
RF	0.94	0.95	0.92	0.93	0.92	0.96	0.94
LR	0.96	0.96	0.97	0.96	0.97	0.96	0.96
ETC	0.94	0.95	0.92	0.93	0.92	0.95	0.94
MLP	0.95	0.95	0.95	0.95	0.95	0.95	0.95
AC	0.95	0.95	0.95	0.95	0.95	0.95	0.95
SVC	0.96	0.95	0.98	0.96	0.98	0.95	0.96
SGDC	0.96	0.96	0.97	0.96	0.96	0.96	0.96

**Table 8:** Accuracy, precision, recall and F1-score results of all ML models using BoW.

Model	Accuracy	Negative class			Positive class		
		Precision	Recall	F1-Score	Precision	Recall	F1-Score
RF	0.94	0.96	0.92	0.94	0.92	0.96	0.94
LR	0.96	0.96	0.97	0.96	0.97	0.96	0.96
ETC	0.94	0.96	0.92	0.94	0.92	0.96	0.94
MLP	0.96	0.96	0.96	0.96	0.96	0.96	0.96
AC	0.96	0.95	0.96	0.96	0.96	0.95	0.96
SVC	0.96	0.96	0.96	0.96	0.96	0.96	0.96
SGDC	0.96	0.95	0.96	0.96	0.96	0.95	0.95

### Performance comparison of ML models using TF-IDF, BoW and GloVe

**Figure 3:**
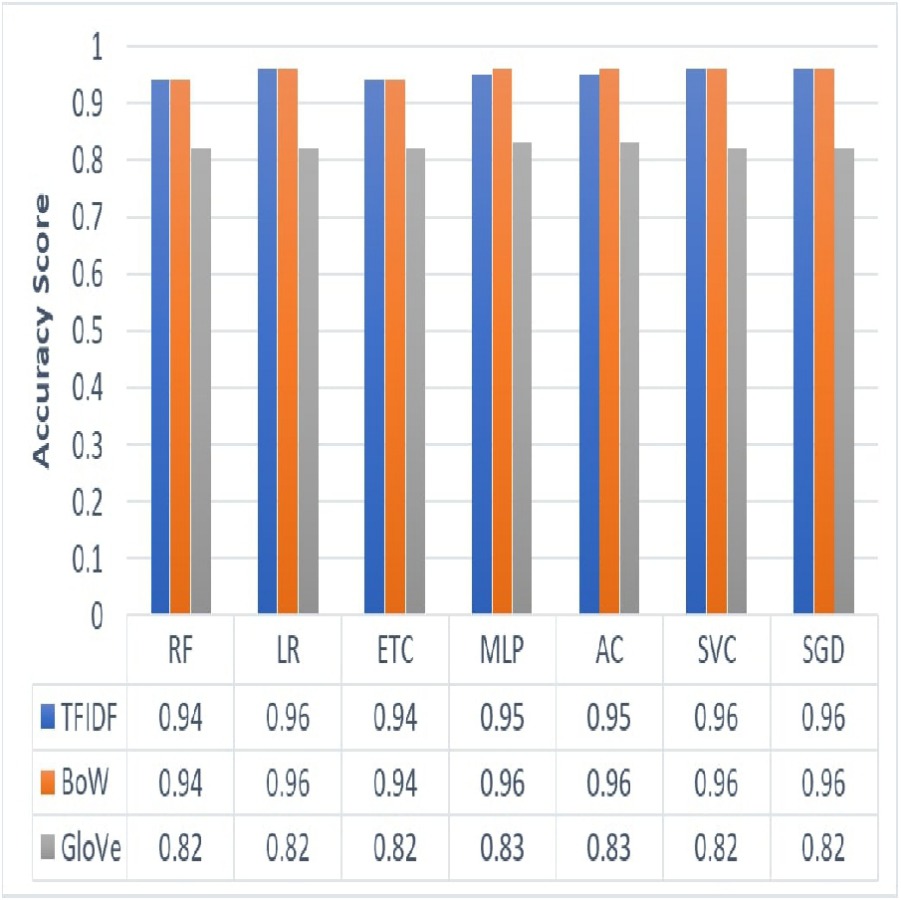
Graphical representation of accuracy results yielded by ML models using TF-IDF, BoW and GloVe.

We employed three feature extraction techniques to analyze the most appropriate technique for the classification of employees’ reviews data under analysis. Figure 3 shows results yielded by the proposed approach using three different feature extraction techniques such as TF-IDF, BoW, and GloVe. We can see that the overall performance of BoW is observed to be best as compared to TF-IDF and GloVe. BoW tends to produce a feature set based on word count regardless of the grammar or structure of a sentence. TF-IDF produces weighted features and eliminates common words thus reducing feature set size. On the other hand, GloVe extracts features by generating a co-occurrence matrix which sometimes is not able to learn words that are out of vocabulary. This causes a reduction in the accuracy of classification results. In terms of ML models, LR with its effective and efficient architecture along with SVC and SGD has performed well with TF-IDF and BoW. Similarly, MLP and AC showed maximum accuracy with the BoW feature set. On the other hand, RF, ETC have shown poor performance in the case of all three feature sets of the employees’ reviews data under consideration.

**Table 9:** Accuracy, precision, recall and F1-score results of all ML models using GloVe.

Model	Accuracy	Negative class			Positive class		
		Precision	Recall	F1-Score	Precision	Recall	F1-Score
RF	0.82	0.81	0.84	0.82	0.83	0.80	0.82
LR	0.82	0.81	0.83	0.82	0.83	0.81	0.82
ETC	0.82	0.81	0.84	0.82	0.83	0.81	0.82
MLP	0.83	0.81	0.87	0.84	0.86	0.80	0.83
AC	0.81	0.82	0.81	0.81	0.81	0.82	0.81
SVC	0.82	0.82	0.84	0.83	0.83	0.81	0.82
SGDC	0.82	0.84	0.81	0.82	0.81	0.84	0.82

**Figure 4:**
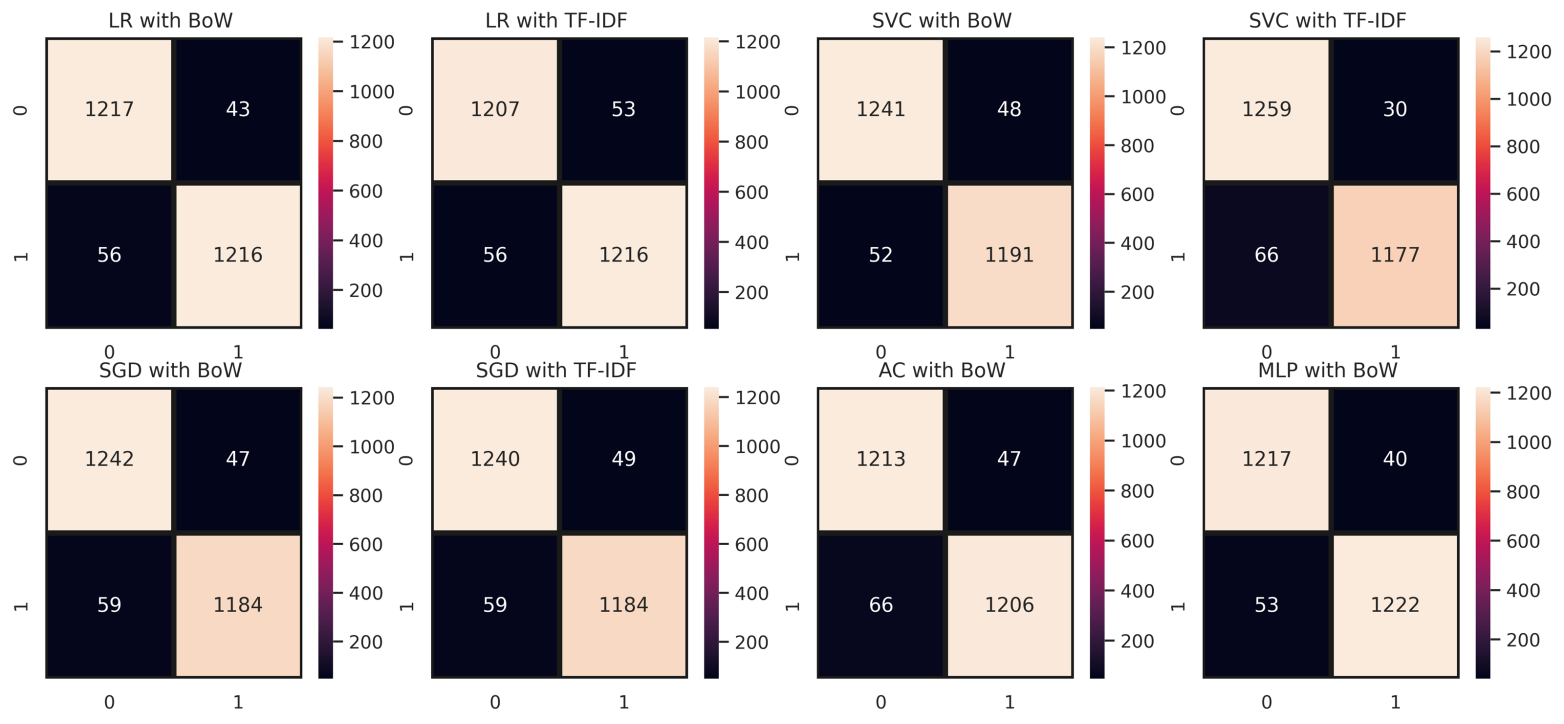
Confusion matrices of ML models with maximum accuracy (0: Negative Class; 1: Positive Class).

Figure 4 demonstrates the confusion matrix of ML models with maximum accuracy for classification of employees’ reviews data as positive and negative. We can see that LR with BoW predicted a total of 2,433 correct instances among 2,532 total instances in which 1217 instances were correctly predicted from negative class and 1216 instances were correctly predicted from positive class. Whereas, it predicted 43 instances as negative which belonged to the positive class and 56 instances belonging to the negative class were predicted positive. There can be seen a minor difference in the performance of LR with TF-IDF as it predicted 2,439 correct instances among a total of 2,532 instances. It succeeds in predicting positive instances more accurately. As for MLP and AC, we can see that they lagged behind LR in classifying employees’ reviews correctly as positive or negative. MLP predicted 2,423 correct instances and AC predicted 2,419 correct instances which are somewhat less than instances correctly predicted by LR. In the case of SVC, it predicted a total of 2,436 correct predictions and 96 wrong predictions with TF-IDF whereas, with BoW it predicted 2,432 correct predictions and 100 instances were predicted wrong which demonstrates that SVC works comparatively better with TF-IDF. In the case of SGD, out of 2,532 instances, it predicted 2,426 correct instances, and 106 instances were predicted wrong in terms of BoW features. Conversely, SGD with TF-IDF carried out 2,424 correct predictions and 108 wrong predictions showing that it performed better with weighted features of TF-IDF. In summary, we can say that LR, SGD, and SVC with TF-IDF performed comparatively better in our proposed approach which is the base of our proposed voting classifier.

### Performance analysis of proposed RV-SGDC

The proposed model RV-SGDC outperforms all other individual models in terms of all evaluation parameters. The study performed experiments in the same environment using RV-SGDC as we did with individual models and find the ensemble model is significant with all features. RV-SGDC results are shown in Table 10 and according to the results, the model achieved the highest accuracy of the study 97% with the TF-IDF features. This significant performance shows the efficiency of the proposed ensemble architecture and weighted TF-IDF features. RV-SGDC also achieved the highest F1 score on both negative and positive target classes with TF-IDF features.

**Table 10:** Accuracy, precision, recall, and F-score results of proposed RV-SGDC model with TF-IDF, BoW and GloVe.

Features	Accuracy	Positive class			Negative class		
		Precision	Recall	F1-Score	Precision	Recall	F1-Score
TF-IDF	0.97	0.97	0.96	0.97	0.96	0.98	0.97
BoW	0.96	0.96	0.96	0.96	0.96	0.97	0.96
GloVe	0.84	0.83	0.82	0.83	0.83	0.84	0.83

**Figure 5:**
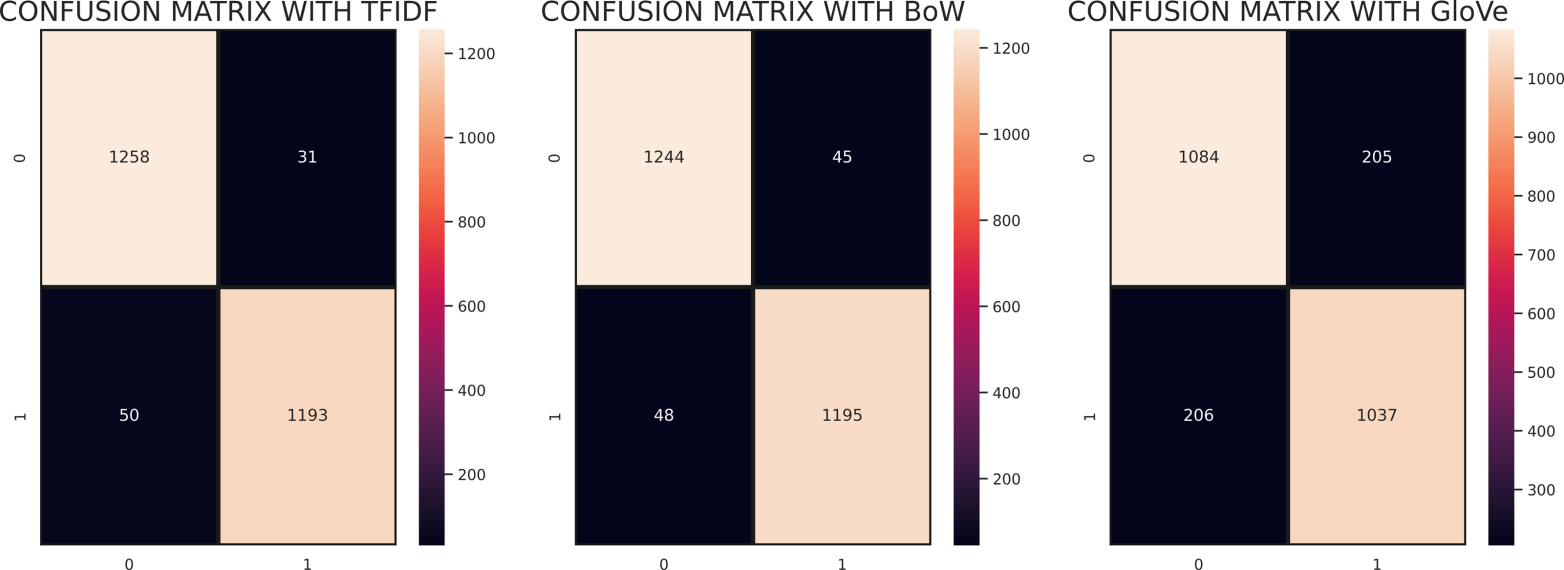
Confusion matrix of RV-SGDC with TF-IDF, BoW, and GloVe features.

Figure 5 shows the confusion matrix for the evaluation of RV-SGDC with TF-IDF, BoW, and GloVe features. According to the confusion matrices, RV-SGDC gave the lowest wrong predictions ratio of the study with TF-IDF features which are 81 out of 2532 and gave 2451 correct predictions. Performance with BoW feature was also good in comparison to GloVe which gave 103 wrong predictions out of 2532 predictions and 2429 correct predictions. RV-SGDC also gave the highest correct prediction ratio using GloVe features compared to other individual models. RV-SGDC gave 2121 correct predictions out of 2531 using GloVe features which show the significance of proposed models on all features.

Further for performance analysis, we have experimented 15 times, each time we run the train and test split method to train and test models on different sets. The mean accuracy and standard deviation (SD) of each model after 15-time experiments are shown in Table 11. There is not too much fluctuation in the results with that method. Still, the RV-SGDC is on top with 0.96 mean accuracy 0.01, 0.02 SD with TF-IDF and BoW respectively. There is some fluctuation with GloVe features as RV-SGDC accuracy goes down 0.84 to 0.82 with 0.02 SD.

**Table 11:** Mean accuracy and SD after doing experiments 15 times on different training and testing sets.

Model	TF-IDF		BoW		GloVe	
	Accuracy	SD	Accuracy	SD	Accuracy	SD
RF	0.92	0.02	0.91	0.03	0.80	0.03
LR	0.95	0.01	0.93	0.02	0.82	0.02
ETC	0.93	0.03	0.92	0.05	0.81	0.04
MLP	0.93	0.02	0.94	0.03	0.80	0.02
AC	0.91	0.05	0.92	0.07	0.81	0.07
SVC	0.94	0.02	0.93	0.03	0.81	0.02
SGDC	0.94	0.01	0.95	0.02	0.82	0.03
RV-SGDC	0.96	0.01	0.96	0.02	0.82	0.02

K-fold cross-validation is also applied to classify employee reviews and analyze the performance of RV-SGDC. For the experimental process, 10 fold were used. Experimental results of ML models with 10-fold cross-validation using TF-IDF features demonstrate that Rv-SGDC performs well with a maximum accuracy score of 0.96. Whereas LR, SVC, SDGC, and AC also perform well with 0.95 accuracies. The performance of all models shown in Table 12.

### Performance comparison of models with another dataset

In this section, the results of all models on another dataset are presented to make a comparison. The US airlines tweets dataset is used to perform the experiments to show the significance of the proposed approach. TF-IDF feature extraction technique is used to perform analysis. Table 13 shows the performance results of all used models on the US airline tweets dataset.

**Table 12:** Ten fold cross-validation results with TF-IDF.

Model	Accuracy
RF	0.91 (+/- 0.11)
LR	0.95 (+/- 0.06)
ETC	0.91 (+/- 0.11)
MLP	0.94 (+/- 0.07)
AC	0.95 (+/- 0.06)
SVC	0.95 (+/- 0.06)
SGDC	0.95 (+/- 0.06)
RV-SGDC	0.96 (+/- 0.05)

**Table 13:** Accuracy, precision, recall and F1-score results of all ML models using TF-IDF.

Model	Accuracy	Negative class			Positive class		
		Precision	Recall	F1-Score	Precision	Recall	F1-Score
RF	0.88	0.89	0.79	0.84	0.87	0.94	0.90
LR	0.90	0.91	0.84	0.87	0.89	0.94	0.92
ETC	0.88	0.91	0.79	0.85	0.87	0.94	0.91
MLP	0.89	0.87	0.85	0.86	0.90	0.91	0.90
AC	0.89	0.90	0.82	0.86	0.88	0.94	0.91
SVC	0.89	0.91	0.82	0.86	0.88	0.94	0.91
SGDC	0.89	0.90	0.84	0.87	0.89	0.94	0.92
RV-SGDC	0.90	0.91	0.83	0.87	0.89	0.94	0.92

### Performance comparison of RV-SGDC with previous studies

The performance of the proposed framework was compared to previous studies carried out to classify sentiments of employees’ reviews. The author proposed multilayer perceptron (MLP); a deep neural network; for classification of employees’ reviews as satisfied (positive) and unsatisfied (negative) in Rustam et al. (2021a). They integrate overall numeric rating (1-5) given by employees as the ground truth for training and testing of ML models. Reviews with an overall rating greater than or equal to 2.5 were considered as satisfied reviews and the rest were considered as unsatisfied reviews.

In Rehan et al. (2021) researcher’s proposed a framework based on combining of two modules by AND gate in which averaging the numeric ratings were considered for classification of reviews as positive and negative in module 1 and module, 2, summary and overall ratings were used as features and classes respectively for classification tasks. Furthermore, the outputs from these modules are unified to classify the reviews as proper and improper. Since this study is concerned with classifying employees’ review data into positive (satisfied) and negative (unsatisfied) therefore; we will compare our results with module 2.

The study uses polarity-based sentiments of employees. In this study, TextBlob is utilized for extracting the sentiment scores of the reviews which are further categorized as positive and negative sentiments based on the threshold value. Whereas Rustam et al. (2021a) used the overall rating of employees. In this research work to analyze the sentiments of TextBlob and overall rating, the dataset is manually assessed which shows that TextBlob produces more accurate results as compared to the overall rating because in overall rating if the rating is positive and text is negative then it is labeled as positive as well as if the text is positive and the rating is negative then it is also labeled as positive and same for the negative label. So, the dataset is analyzed manually to check the ground truth. Results in Table 14 show that the current study outperformed previous studies in text classification of employees’ reviews as positive and negative. The proposed system performs best from previous studies by using RV-SGDC. RV-SGDC performs best because of its ensemble architecture.

### Statistical analysis to show significance of RV-SGDC

This study performs a *T*-test to show the statistical significance of the proposed RV-SGDC model on other used state-of-the-art models (Fatima et al., 2021). *T*-test gives us output in terms of acceptance or rejection of the null hypothesis.

• Null Hypothesis: The proposed model is statistically significant as compared to other models.
• Alternative Hypothesis: The proposed model is not statistically significant as compared to other models.

RV-SGDC accepts the null hypothesis with TF-IDF features in comparison with other models which show that the model is statistically significant. While in the case of the BoW case rejects the null hypothesis and accepts the alternative hypothesis which means that RV-SGDC is not too statistically significant on BoW features. In the case of GloVe features, RV-SGDC shows statistical significance on other models by accepting null hypotheses.

**Table 14:** Performance comparison of previous studies with current study.

Ref	Dataset	Features	Ground truth	Features	Model	Results
	Employees’ Reviews	Summary	Overall Rating	TF-IDF	MLP	0.83
	Employees’ Reviews	Summary	Overall Rating	TF-IDF	ETC	0.79
Proposed	Employees’ Reviews	Summary, Pros, Cons, and Advice to Management	TextBlob Sentiments	TF-IDF	RV-SGDC	0.97

## Conclusion

This study experimented with sentiment classification using lexicon and machine learning based techniques. Analysis was performed on employee reviews datasets which contains six well-known companies’ employees’ reviews to find the sentiments using text review. First, this study used preprocessing techniques to clean the text dataset, and then TextBlob was used to label the data with sentiments. TF-IDF, BoW, and GloVe features are used to train machine learning models. Performance of machine learning classifiers such as RF, LR, ETC, MLP, SVC, SGD, AC, and proposed RV-SGDC analyzed on these text features for the sentiment classification. Obtained results show that RV-SGDC attained a maximum accuracy score of 0.97 with TF-IDF features followed by similar precision, recall and f1-score whereas from BOW and GloVe RV-SGDC achieved 0.96 and 0.84 respectively. This significant performance of RV-SGDC shows the efficiency of the voting scheme between multiple models for final prediction. TF-IDF also shows its significance because of its approach to compute weighted features for machine learning models. Moreover, the proposed approach is compared with previous work conducted in the same direction. Results show that the proposed approach yielded state-of-the-art results as compared to previously done research on the same dataset concerning sentiment classification of employees’ reviews. It can be observed that using TextBlob sentiments has highly impacted the accuracy of ML models individually as well as in a voting classifier as an improvement of $\widetilde {0}$.14 accuracy score is observed with our experiments. In the end, we also show the statistical significance of the proposed model RV-SGDC on other models using the *T*-test which shows that the voting model is statistically significant as compared to individual models using TF-IDF features. However, the obtained results are not irrefutable as accuracy can be impacted by integrating a larger dataset which is the future direction of this work. One of the possible future directions can be the integration of deep learning models for improvement in accuracy results. We will consider ensemble deep learning models such as CNN-LSTM with the latest embedding schema which can be useful to improve the results.

## Supplemental Information

10.7717/peerj-cs.712/supp-1 Supplemental Information 1 Experimental file

## Additional Information and Declarations

### Data Availability

The following information was supplied regarding data availability: The Supplemental File contains the experiment in Python using a machine learning approach. The dataset is available at Kaggle: https://www.kaggle.com/saqlainrehan/employeesreviews-dataset.
